# Editorial: Symmetry in protein architecture: Evolution, design, structure-function relationship, and applications

**DOI:** 10.3389/fmolb.2023.1140877

**Published:** 2023-01-23

**Authors:** Michael Blaber, Suman Kundu

**Affiliations:** ^1^ Biomedical Sciences, Florida State University, Tallahassee, FL, United States; ^2^ University of Delhi, New Delhi, India

**Keywords:** protein evolution, protein design, protein folding, protein function, protein assembly

Meiering et al. (“Engineering the kinetic stability of a β-trefoil protein by tuning its topological complexity”) address a novel area of protein design (the targeted engineering of unfolding kinetics) using the threefold symmetric β-trefoil architecture as the model system. A *de novo* designed purely-symmetric β-trefoil protein (“Threefoil”) was found to have enhanced kinetic stability through reduced unfolding kinetics. This is an attractive property for engineered proteins as it can reduce proteolysis and increase functional half-life. The authors focus upon the contact order of core residues in the β-trefoil architecture since the Threefoil protein has more extensive contacts in comparison to a naturally-evolved, less-symmetric (and faster unfolding), β-trefoil protein (hisactophilin). In this report the authors show that core-substitution mutations that make the hisactophilin core more like that of the Threefoil protein (i.e., increasing contact order) results in greater kinetic stability (i.e., slower unfolding kinetics). The threefold symmetry of the β-trefoil architecture provides the possibility of multiple instances of such mutations, and limiting mutations to the core region minimizes potential negative effects upon function (which typically involve surface positions).

Tamada et al (“Creation of Cross-Linked Crystals With Intermolecular Disulfide Bonds Connecting Symmetry-Related Molecules Allows Retention of Tertiary Structure in Different Solvent Conditions”) describe manipulation of the macromolecular symmetry involving crystal contacts. Protein crystals can provide diffraction data, yielding atomic-resolution structures. Furthermore, the solvent content of protein crystals often enables substrate diffusion, and the molecular motions necessary for enzyme function; thus, crystallization of enzymes can yield corresponding structure and function data. However, intermolecular crystal contacts in proteins often involve solvent molecules, and are thereby disrupted by alterations in the pH, salt, and additive conditions of their crystallization solutions. Residue positions participating in intermolecular crystal contacts are occasionally appropriately juxtaposed to permit substitution by cysteine residues, thereby permitting formation of covalent (i.e., disulfide bonds) between such crystal contacts. Tamada et al, working with crystals of phage T4 lysozyme, introduce a series of such cysteine mutations, followed by oxidation to cystine after crystallization. They show that such mutations permit radical alterations in buffer conditions of the crystal, even including substitution into organic solvents, all the while retaining atomic resolution diffraction properties.

Prakash and Gosavi (“The diversity of protein-protein interaction interfaces within T = 3 icosahedral viral capsids”) discuss another manifestation of macromolecular symmetry, namely, the remarkably complex twofold, threefold, fivefold and sixfold (quasi-equivalent) symmetry of the coat proteins of icosahedral viruses. Assembly and disassembly of such viral capsids is critical for viral maturation and infectivity; however, such processes are poorly understood. These authors characterize the relative strengths of the different coat protein interface symmetries by quantifying their size and hydrophobicity. The authors report distinct differences in the dimerization interface of coat proteins from different viruses, but also, that, in general this interface is the strongest when compared to the threefold, fivefold and sixfold interfaces. The results provide insight into the likely hierarchical assembly/disassembly of such viral proteins.

Blaber (“Variable and Conserved Regions of Secondary Structure in the β-Trefoil Fold: Structure Versus Function”) provides an analysis of the β-trefoil family of proteins from the standpoint of what region is structurally conserved, and what region(s) are divergent among the various family members. This protein architecture has pseudo-threefold rotational symmetry, where the repeating motif (the “trefoil” motif) is described by a pair of anti-parallel β-strands of approximately 40–50 amino acids in length. An analysis of this diverse and functionally-divergent family shows that there is a conserved fundamental structure comprising the essential β-strands, and that the variability among family members is essentially limited to variability within loop/turn structures between various β-strands. This apparent structure/function segregation provides insight into design strategies for novel β-trefoil proteins, as well as evolutionary processes associated with emergence of the β-trefoil *via* duplication/fusion events that can create novel functionality.

Although several of the common symmetric protein architectures are represented by this Research Topic (i.e., the β-propeller, β-trefoil, icosahedral viral capsid proteins), some other important symmetric protein architectures that have been the subject of much study (i.e., TIM-barrel, linear repeat/solenoid proteins) are not represented. There is, therefore, many additional aspects of symmetry in protein architecture yet to be discussed.

